# Wet, volatile, and dry biomarkers of exercise-induced muscle fatigue 

**DOI:** 10.1186/s12891-016-0869-2

**Published:** 2016-01-21

**Authors:** Josef Finsterer, Vivian E. Drory

**Affiliations:** Krankenanstalt Rudolfstiftung, Postfach 20, 1180 Vienna, Austria; Medical Center, Tel-Aviv, Israel

**Keywords:** Biomarker, Biological marker, Muscle fatigue, Monitoring, Muscle exercise, Exercise fatigue, Exhaustion, Endurance

## Abstract

**Background:**

The physiological background of exercise-induced muscle fatigue(EIMUF) is only poorly understood. Thus, monitoring of EIMUF by a single or multiple biomarkers(BMs) is under debate.

After a systematic literature review 91 papers were included.

**Results:**

EIMUF is mainly due to depletion of substrates, increased oxidative stress, muscle membrane depolarisation following potassium depletion, muscle hyperthermia, muscle damage, impaired oxygen supply to the muscle, activation of an inflammatory response, or impaired calcium-handling. Dehydration, hyperammonemia, mitochondrial biogenesis, and genetic responses are also discussed. Since EIMUF is dependent on age, sex, degree of fatigue, type, intensity, and duration of exercise, energy supply during exercise, climate, training status (physical fitness), and health status, BMs currently available for monitoring EIMUF have limited reliability. Generally, wet, volatile, and dry BMs are differentiated. Among dry BMs of EIMUF the most promising include power output measures, electrophysiological measures, cardiologic measures, and questionnaires. Among wet BMs of EIMUF those most applicable include markers of ATP-metabolism, of oxidative stress, muscle damage, and inflammation. VO_2_-kinetics are used as a volatile BM.

**Conclusions:**

Though the physiology of EIMUF remains to be fully elucidated, some promising BMs have been recently introduced, which together with other BMs, could be useful in monitoring EIMUF. The combination of biomarkers seems to be more efficient than a single biomarker to monitor EIMUF. However, it is essential that efficacy, reliability, and applicability of each BM candidate is validated in appropriate studies.

## Background

A biomarker (BM) is a measurable molecule or test, to objectively monitor the change of a condition or process over time, after treatment, or after training [1]. BMs may be used for diagnostic purposes, for monitoring purposes, or for risk assessment [2]. BMs can be used as an indicator of a biological state, or to objectively assess a biological or pathological process. To assess the degree of muscle fatigue (continuous reduction of muscle force (maximal voluntary contraction (MVC)) during exercise (exercise-induced muscle fatigue (EIMUF), peripheral fatigue) and the impact of exercise on the skeletal muscle, various BMs have been proposed but their reliability is questionable [3]. BMs of EIMUF need to be differentiated from BMs of physical fitness, muscle damage (injury), of overtraining, of inflammation, and from BMs of central fatigue (central nervous system disease) or fatigue due to systemic disease (anemia, menstruation, malignancy, cardiac disease, chronic infection, vitamin deficiency, or hepatopathy). To approach the topic of monitoring EIMUF it is essential to establish a definition of EIMUF and to elucidate its physiologic background. Aim of this review was to describe and discuss established findings and new aspects emerging from the clinical and experimental literature about BMs of EIMUF. These new aspects are expected to influence experimental modelling, development of diagnostics, and development of new BMs to more accurately monitor EIMUF.

## Method

Data for this review were identified by searches of MEDLINE, Current Contents, Springerlink, Wiley, EBSCO, Ovid, and Web of science by applying a sensitive search strategy using combinations of the following search terms: “biomarker”, “biological marker”, in combination with “fatigue”, “fatigability”, “muscle”, “exercise”, “exercise-induced”, and “exhaustion”. Further manual search was conducted to identify other relevant articles from cross-references. Randomized (blinded or open label) clinical trials, longitudinal studies, case series, and case reports were considered. Abstracts and reports from meetings and animal studies were not included. Only articles about humans and published in English, French, Spanish, or German between 1966 and 2015 were considered. Appropriate papers were studied and discussed for their suitability to be incorporated in this review.

## Results

Altogether 134 papers were found to be suitable to match with the intentions of the review. Of these, 91 were selected, because they were available as full papers, they provided most recent data, they provided an extensive, in-depth discussion of their results, and they compared previous findings with the current results.

### Biomarkers

#### Requirements a BM must meet

Not each molecule or test is eligible to serve as an appropriate BM of EIMUF. Requirements a BM must meet are that the correlation between the measurement and the represented process is linear, that it shows the continuous change of the process, and that it appropriately reflects the outcome of a condition in the sense of a linear outcome measure. Additionally, BMs must register quick changes (translational marker) and need to change as a function of the process being monitored. They need to be stable without appreciable diurnal variations, need to correlate significantly with exercise intensity, and need to be present in detectable amounts in easily accessible biological fluids [2]. It is also essential that the usefulness and efficiency of a BM has been validated and proven, not to unnecessarily increase costs. Generally, BMs need to be easily collected (easily accessible tissues), cheap, the test should be easy to perform with widely available equipment, should be reliable when tested by different examiners and repeatedly, and independent of age, sex, environmental and climate conditions, pre-existing training condition, food, hydration, daytime, and the exercise monitored. It is also important that the test carried out to monitor EIMUF does not further induce fatigue. Most of the available BMs of EIMUF do not meet these requirements [3].

#### Dependency of a BM

Since there is no ideal BM, BMs depend on various different factors. These include age, sex, degree of general fatigue, type, intensity, frequency, and duration of exercise (e.g. voluntary or electrical stimulation), type of contraction (isometric, isotonic, intermittent, continuous), environmental climatic conditions (temperature, humidity, wind speed), food, daytime. and physiological and training status (physical fitness) of an individual [1, 4]. A BM may also depend on the energy supply during exercise, on hydration, and on the health status (healthy or diseased individuals) [5]. The optimal choice of a BM is not only dependent on the factors just mentioned but also on how good the physiology of the process to be monitored is understood.

#### Classification of BMs

**Fig. 1:**
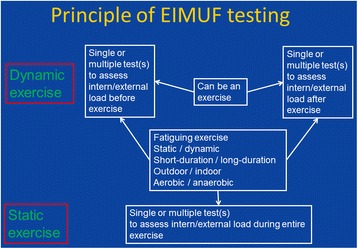
General test arrangement to monitor EIMUF. Monitoring tests may be generally carried out before and after the test (usually for dynamic exercise) or continuously during the entire test (usually for static exercise)

BMs can be classified according to the method applied as wet, volatile, or dry BM [6], as translational or non-translational BM, as invasive or non-invasive BM, as validated or non-validated BM, or as BM for measuring focal or generalised EIMUF. Most wet BMs derive from blood, saliva, or urine. Only rarely other body fluids are investigated. Most dry BMs are measures of the power output, cardiac parameters, or scales or questionnaires. Needle and particularly surface electromyography (EMG) are also applied for monitoring EIMUF. The choice of a BM for monitoring EIMUF depends on the duration, type, and intensity of exercise performed. There is a difference if the exercise is short-lasting for only a few seconds or minutes or a long-lasting condition, like in marathon runners or triathloners. It is also strongly dependent on the type of contraction. BMs can be also categorised according to the exercise performed, which can be either assessed by another test or can be the BM itself (Fig. 1). BMs of EIMUF may be further differentiated into external and internal BMs. External load quantifying tools include power output measuring devices, time-motion analysis (to analyse movement patterns in team-sport), or movement kinematics or kinetics, whereas internal load quantifying tools include, for example, cardiac parameters, neuromuscular function, biochemical, hormonal, or immunological assessments, and questionnaires (Table 1) [3].

**Table 1:** BMs of EIMUF currently under discussion

Dry BMs	
Power output	
Measurement of muscle force	
Counter movement jump	
Cycle ergometer sprint test	
Running speed	
Tongue pressure	
Electrophysiological tests	
Surface-EMG	
Muscle torque	
M-wave duration	
Nerve conduction velocity	
Transcranial magnetic stiumulation	
Cardiac parameters	
Heart rate	
Movement kinematics and kinetics	
Myo-mechanogram	
Scales and questionnaires	
Wet BMs	
ATP metabolism	
Lactate	
Oxipurines	
Ammonia	
Oxidative stress	
TBARS	
GSH	
TAC	
Immune response	
Leukocytes	
Cortisol	
Interleukines	
Muscle damage	
C-terminal agrin fragment	
Volatile BMs	

### Classification of exercise

Exercise, carried out to induce fatigue, may be classified according to various criteria such as duration, intensity, or type of muscle contraction or according to the type of energy production (aerobic or anaerobic). According to duration and intensity, exercise may be classified as short-duration, low-intensity exercise, as short-duration, high intensity exercise, or as long-duration exercise. According to the type of energy production exercise may be classified as <20 sec in duration (up to 90 % anaerobic energy), 20 sec to 1 min (aerobic and anaerobic energy), and exercise lasting >1 min (>50 % aerobic energy). It is essential to know that exercise may not induce fatigue if exercise is mild and carried out for a very short time. Exercise may be also classified as static (no movement of entire body) or as dynamic (movement of entire body (e.g. sport)) [7]. Exercise may be carried out within an experimental setting (e.g. standardised ergometer (simulated)) or as an open-air exercise (running, walking (real)) [8]. Muscle contraction during exercise may be voluntary or stimulated, continuous or discontinuous, or isometric (no change of length), isotonic (muscle length changes) which can be either eccentric or concentric, or isokinetic (like isotonic but with constant speed).

### Exercise-induced muscle fatigue (EIMUF)

EIMUF is a complex and multimodal process involving various metabolic and functional mechanisms.

#### Definition of EIMUF

Due to the uncertainties about the complex nature of EIMUF a number of different definitions exist depending on the underlying experimental model or the conditions under which EIMUF develops [3]. One of the most commonly applied definitions of fatigue describes EIMUF as “a failure to maintain the required or expected muscle force” (reduced power output (work rate), ATP turnover rate) [9]. In this definition fatigue is regarded as decrement of power production (decline of muscle force, decreased force output) and EIMUF corresponds to the reduction of muscle force during exercise [10, 11]. Another popular and more general definition of EIMUF defines it as “inability to complete a task that was once achievable within a recent time frame” [3]. Exercise scientists define EIMUF as “exercise-induced impairment of muscle performance” [10]. Exercise intensity is initially sustained by recruitment of new motor units and help from synergistic muscles [10]. As exercise proceeds, technique and motor skill execution deviates to maintain outcomes but lastly they deteriorate resulting in reduced accuracy and velocity [10]. EIMUF may be also defined as divergence between external load (work completed by a subject and measured independently of its internal characteristics) and internal load (relative physiological or psychological stress imposed) [3]. A combination of both may be important for monitoring EIMUF [3]. An example for an external load is the power output sustained for a given duration. Examples for internal loads are the heart rate or perception of the effort [3]. Compensatory mechanisms of fatigue are an altered CNS muscle recruitment pattern, recruitment of additional motor units, synchronisation of firing frequencies, and increase of firing frequencies. Fatigue may be physiologic or pathologic. It may last for a short while or for days (e.g. 6 days in soccer players after a match) [12]. Fatigue is dependent of similar factors as described for BMs [3].

#### Physiology of muscle fatigue

To understand and monitor EIMUF it is essential to include all physiological aspects of EIMUF. Currently, however, the physiology of EIMUF is not fully understood because it is difficult to investigate an active muscle and because several processes run off simultaneously during any type of muscle exercise [13]. Thus, it is essential for future studies to find out which changes and processes contribute most to EIMUF. Presumably responsible for EIMUF are a shortage of substrates to maintain contraction, such as carbohydrates, calcium, sodium, potassium, or ATP, continuous oxidative stress, electrophysiological changes due to decrease of membrane depolarisation, hyperthermia, muscle damage, inflammatory processes, calcium-handling, and impaired oxygen supply (hypoxia) of the contracting muscle [14, 15].

##### Depletion of carbohydrate deposits

During a marathon there is progressive depletion of carbohydrate reservoirs (glucose deposits in the liver and muscle) due to the active muscle [14, 16]. Insufficient supply of carbohydrates during a marathon can lead to hypoglycemia and consecutively muscle fatigue [17]. In addition to carbohydrates other essential metabolites, such as ascorbic acid, calcium, sodium, potassium, and ATP may be depleted during exercise [7]. Depletion of ATP may be associated with an increase in ADP, which inhibits sodium/potassium ATPases and calcium-ATPases [18].

##### Production of reactive oxygen species (ROS)

Exercise induces excessive production of ROS leading to oxidative stress, fatigue and reduced exercise performance [19, 20]. Oxidative stress is highest immediately after exercise but may rise again hours after finishing exercise [2]. Accordingly, oxidative stress parameters are typically increased immediately after exercise [21]. Oxidative stress in form of ROS is implicated in the damage of various macromolecules, immune dysfunction, and muscle damage [19]. Oxidative stress may not only be responsible for EIMUF but also for impaired recovery from exercise [22].

##### Membrane depolarisation due to potassium depletion

During high-intensity, long-term exercise potassium is continuously leaking from the muscle cell decreasing the membrane potential and thus depolarising the muscle membrane, therefore currents cannot propagate along the membrane or get into the triad [23]. Consecutively, the muscle becomes weak [1, 24]. Additionally, depletion of potassium from muscle cells results in progressive hyperkalemia with increasing exercise intensity [25].

##### Hyperthermia

Particularly after long-duration exercise hyperthermia (exertional heat illness, core temperature >39°) develops, reducing muscle performance [14, 21]. Interestingly, those with high post-exercise core temperature maintain a steady pace throughout the exercise while those with a lower post-exercise core temperature show lower pace at the end of the run [14]. Thus, post-race temperature correlates positively with mean running pace [14]. This could be due to increased muscle damage among those with low pace and thus lower core temperature [14].

##### Muscle damage

Muscle damage is another factor contributing to muscle fatigue during endurance activity. In a study of 25 triathlon participants there was a positive correlation between reduced muscle performance and blood markers of muscle damage, such as creatine-kinase (CK), lactate-dehydrogenase (LDH), or myoglobin [26]. This study further showed that there is a relation between muscle damage and fatigue [26]. Accordingly, marathoners with higher levels of running fatigue, develop higher levels of CK, LDH, and myoglobin compared to marathoners with low levels of running fatigue [14]. Another study found a positive correlation between post-race urinary myoglobin concentration, an indirect marker of muscle damage, and the decrease of muscle performance after a marathon [27]. When performing muscle biopsy before and after a marathon, it has been evidenced that a marathon produces muscle fiber necrosis and inflammation [28]. These morphological alterations are similar to those found in rhabdomyolysis [28]. These findings suggest that muscle fatigue, particularly during endurance exercise, may partly derive from muscle damage. Muscle damage is one of the most relevant sources of EIMUF in long-distance runners [26].

##### Cardiac stress

Cardiac stress as measured by the cardiac stress index (CSI), using a non-linear, detrended fluctuation analysis (DFA) of the heart rate, was positively correlated with the Borg rating perceived exertion (RPE) scale suggesting that cardiac stress contributes to EIMUF [29]. Since the CSI effectively quantifies the degree of cardiac stress during exercise, it can be used to monitor the cardiac condition during physical exercise [29]. Additionally, it has been shown in Sprague–Dawley rats that heart failure speeds up the microvascular pO_2_ mean response time (blood-muscle O_2_ driving gradient) [30]. Conversely, in the soleus muscle of rats low microvascular pO_2_ reduces the blood-muscle O_2_ driving gradient [30].

##### Inflammation

Inflammatory processes are induced during exercise as a result of muscle metabolism and muscle damage [22]. Muscle biopsy from participants after a marathon showed muscle fiber necrosis and inflammation [28]. Classical BMs of inflammation are the blood leukocytes, C-reactive protein, interleukines, and serum or salivary cortisol [22].

##### Calcium handling

There are some indications that impaired calcium handling contributes to the development of EIMUF [31]. Sarcalumenin is a Ca-binding protein of the sarcoplasmatic reticulum in muscle cells [31]. Sarcalumenin knock-out mice have an enhanced fatigue resistance [31], suggesting that the expression of the gene is involved in the muscle fatigue mechanism.

##### Mitochondrial dynamics

Excessive exercise increases the muscle atrophy marker atrogin-1 and MuRF-1 mRNA, which is accompanied by increased autophagy and fission of mitochondria in muscle cells [13]. Excessive exercise also decreases peroxisome proliferator-activated receptor (PPAR)-γ coactivator PGC-1alpha and complex-I expression. Furthermore, excessive exercise activates JNK and Erh1/2 pathways resulting in induction of p53, p21, and MnSOD expression [13]. These changes in mitochondrial dynamic remodelling may be responsible for muscle damage and damage of immune functions via downregulation of mitochondrial biogenesis and upregulation of autophagy [13].

##### Other potential mechanisms of EIMUF

Other potential factors contributing to the development of EIMUF may be depletion of muscle proteins into the blood [32], reduction of blood and plasma volume (dehydration) as occurring after a marathon [14], acidosis, accumulation of ammonia, elevated serotonin, hypoxia, hyperkalemia, or changes of the genetic profile, particularly in long-duration exercise [10]. For example, EIMUF and reduced endurance may be due to reduced expression of the transcriptional coactivator PPAR-α (PGC-α), of which ectopic expression results in increased number and function of mitochondria and increase in oxidative, fatigue-resistant muscle fibers [33]. In patients with chronic obstructive lung disease (COPD) accumulation of plasma ammonia under conditions of metabolic stress during exercise contributes to muscle fatigue, independent of the ventilatory limitation [34]. In a study of tumour suppressor factor p53 knock-out mice it has been shown that p53 initiates mitochondrial biogenesis in response to contractile muscle activity [35].

#### Postactivation potentiation (PAP)

When discussing peak-force loss after exhaustion it is important to include the phenomenon of PAP [36]. PAP is characterised by enhancement of exercise performance and maintenance of power output after exercise [36, 37]. The physiologic background of PAP is poorly understood. In a study of 22 endurance-athletes undergoing the University of Montreal track test (UMTT), counter-movement jump (CMJ) height improved, peak-power incremented, and athletes maintained their 20 m sprint performance after exhaustion [36]. In athletes who did not develop PAP, reduction of peak-force, vertical stiffness, and higher vertical displacement of the center of mass correlated with serum lactate levels [36]. PAP can be also observed after other types of exercise, such as rebound throws [37].

### Biomarkers of EIMUF

Studying BMs of EIMUF requires performance of a defined exercise and measurement of parameters (BMs) originating from various different mechanisms of EIMUF. Measurement of BMs may be carried out before, during or after exercise (Fig. 1). Parameters of interest may originate from easily accessible body fluids (wet BMs) or from measuring muscle force, speed of exercise, electrophysiological recordings, cardiac recordings, kinesiology and kinetics, myo-mechanogram (MMG), or from questionnaires (dry BMs). Measuring VO_2 max_ and VO_2_ kinetics during exercise provides the basis for volatile BMs of EIMUF. Not each putative BM of EIMUF can be applied to each type of exercise.

#### Dry biomarkers

Dry BMs are least invasive and thus usually the ones most easily determined. Dry BMs include measurement of muscle force (power output) before and after or during a workload, analysis of electrophysiological recordings, assessment of cardiac parameters, and scales or questionnaires.

##### Power output

There are various test assemblies to perform exercise and to assess power output from this workload to determine muscle fatigue.

Direct measurement of muscle force. Measuring muscle force before and after predefined workload is one of the most frequently applied means to measure EIMUF. Loss of power output during exercise may reflect muscle fatigue. Muscle force before and after a given task can be measured in a single muscle, in a group of muscles, or in all muscles. Measuring muscle force may be carried out with dynamometers, power output measuring devices (probes), clinical assessment of muscle force, or more globally with questionnaires. Various power output measuring devices are available. Various types of exercise may precede force measurement [3]. Leg and arm force production is reduced after any exercise but particularly after a marathon [14]. Maximum isometric force-generating capacity (MIFGC) is reduced immediately after eccentric contraction [38]. MIFGC remains depressed 48 h after exercise [38]. External/internal rotation isokinetic torque [39] is reduced after exercise, Power output is also reduced after fatiguing leg press exercise [40]. In this test, power is calculated as the instantaneous product of displacement velocity and applied force, whereas work output is calculated as vertical displacement of the weight plates times applied force [40]. Even peak power, however, may show diurnal fluctuations with higher values in the evening compared to the morning [21]. Force of bulbar muscles in children can be assessed by means of the slurp test [41].

*Tongue pressure.* An example of assessing power output of bulbar muscles is by measuring the tongue pressure. In neuromuscular disorders with bulbar involvement, such as myasthenia, amyotrophic lateral sclerosis (ALS), bulbo-spinal muscular atrophy (SBMA), oculopharyngeal muscular dystrophy (OPMD), myotonic dystrophy type 1 (MD1), or mitochondrial disorders (MIDs), assessment of bulbar functions is mandatory. In a study of 47 patients with SBMA tongue pressure was measured by means of an intra-aortic pressure probe and questionnaires which assessed swallowing [42]. Tongue pressure was reduced in SBMA patients within 3y after disease onset. Tongue pressure was reduced even in patients without dysphagia and repetition of swallowing compensated for tongue weakness in these patients [42]. Tongue pressure was positively correlated with bulbar-related functional scales [42]. Tongue pressure more strongly correlated with muscle strength of the pharyngeal, neck, and upper limb muscles than with lower limb muscles. Though tongue pressure is not validated as a BM of focal EIMUF, it appears a promising monitor of focal EIMUF and weakness of bulbar muscles since power output can be measured over time before and after exercise and correlates well with scales assessing swallowing [42].

*Jumping.* A further possibility to assess the power output is by means of jumping tests. Most frequently applied is the CMJ test which collects and analysis parameters such as mean power, peak velocity, peak force (PF), peak power (PP), peak torque, jump height, flight time, contact time, and rate of force development (RFD) [3, 43, 44]. The test induces only a minimal amount of additional fatigue itself and thus is useful to monitor EIMUF when carried out before and after exercise [3, 45]. After an isokinetic, concentric exercise peak torque and RFD decreased even 24 h after exercise [43]. Jumping performance seems to deteriorate for as long as 72 h post-exercise whereas strength remains unchanged after an acute bout of intense polymetric exercise [46]. Other jumping tests for measuring the power output include the static jump (SJ) test [46], the vertical jump test [47], and the drop jump [47].

*Cycling.* Another possibility to assess the power output is by means of cycling on an ergometer. Most commonly applied is the cycle ergometer sprint test which specifically quantifies the concentric component of the fatigue-induced decrement of force production in muscle, which may be overlooked by the CMJ test [48]. The cycle ergometer sprint test is a method of monitoring EIMUF in endurance or power-team-sport athletes [48].

*Walking.* Walking test represent a frequently applied means to assess the power output. Examples of walking tests are the 6 m walking test, the 400 m walking speed test, the timed-up-and-go test [49], the 12-min self-paced walking test, and stair climbing [49].

*Running.* Another possibility to assess the power output is by measuring running speed or distance. Reduction of running speed is a simple means of indirectly measuring EIMUF but only a global parameter since assessment may also include central fatigue. Decrease of running speed can be particularly observed in long-distance runners (marathoners) [14]. In a study of 22 endurance athletes carrying out the UMTT, the maximum running speed test remained unchanged after the UMTT, possibly due to PAP [36]. Other running tests include the maximum running speed over a distance of 20 m (20 m sprint test) [36, 47], the total high-intensity running distance (THIR) [45], and the repeated sprint ability (RSA) exercise [50]. Most of the power output tests show diurnal fluctuations with higher values in the afternoon compared to the morning [50].

##### Electrophysiological BMs

*Surface EMG analysis*. Applying surface-EMG, an interference pattern is recorded by bipolar surface electrodes positioned over the endplate zone of the muscle of interest [51]. Signals are then passed to an A/D converter, are band-pass filtered, and converted to root mean square (RMS) or mean rectified voltage for EMG amplitude, which is roughly equivalent to the mean rectified value (MRV). Additionally, surface EMG interference patterns may be analysed by fast Fourier transformation for median frequency, mean power output, or mean frequency (MF) over a distinct epoch [8, 52]. In a study of 12 healthy males performing standardised exercise with two different loadings on a knee extension device, post-loading EMG-amplitude was either reduced or increased depending on the type of loading [8]. MRV or RMS are typically increased after exercise. MF and mean power output are typically reduced after exercise [8]. Muscle fatigue is thus characterised by an increase in the EMG interference-pattern amplitude (recruitment of additional motor units, increase in firing frequency, synchronisation of discharges) and a shift of the spectrum to the left [53, 54]. Recovery from muscle fatigue is characterised by a decrease of the EMG-amplitude and a shift of the spectrum to the right [53].

*Muscle torque.* To assess muscle torque, muscle stimulation is performed on the resting muscle via self-adhesive surface electrodes by delivering single rectangular pulses by a constant-current stimulator to the supplying nerve until a torque plateau is observed [8]. Antagonist muscles are not stimulated. The parameters of interest include the maximum isometric torque, the maximum twitch torque (peak torque), and the half-relaxation time [8]. After exercise the peak torque is typically reduced using variable resistance loadings [8]. Reduction of maximum twitch torque is more pronounced in young as compared to old subjects [8].

*M-wave duration.* A further electrophysiological method to monitor EIMUF is the assessment of the M-wave after stimulation of motor nerves. Stimulation electrodes (cathodes) are placed such that a weak stimulation current gives the strongest response [8]. Then the stimulating current is increased in 10 mA steps until a clear plateau in the M-wave amplitude is reached. Thereafter an additional 25 % of stimulation current is applied (supramaximal stimulation) [8]. In a study of 12 healthy males performing standardised exercise on a knee extension device, post-loading peak-to-peak M-wave duration was significantly increased while area and amplitude were decreased after the bout [8].

*Conduction velocity.* In some studies EIMUF resulted in reduction of the nerve conduction velocity, irrespective if the proband carried out eccentric or concentric exercise [55]. In power athletes as well as in endurance athletes the degree of EIMUF correlated negatively with the nerve conduction velocity [52]. Other studies, however, did not confirm these findings [56]. Contrary to nerve conduction velocity, muscle fiber conduction velocity (MFCV) as assessed by surface EMG decreased with EIMUF at least during dynamic exercise [23, 57, 58]. During static exercise by means of fatiguing isometric contractions, on the contrary, MFCV remained unchanged [58].

*Transcranial magnetic stimulation (TMS).* In 8 patients with electrical injury undergoing a 2 min exercise with MVC, TMS revealed prolongation of the silent period and an increase of the area and amplitude of the M-wave response [59]. These effects could be increased if patients were exposed to muscle ischemia induced by a blood pressure cuff to simulate EIMUF [59]. The technique allows differentiation between the central and peripheral contribution to EIMUF [59].

*EMG fatigue threshold.* The EMG fatigue threshold is defined as the exercise intensity an individual can maintain indefinitely without the need to recruit additional motor units, which is associated with an increase in the amplitude of the interference pattern [60]. Recently, a new practical and reliable method to determine the EMG fatigue threshold has been introduced [60]. The physical working capacity at fatigue threshold (PWCFT) is defined as average of the highest power output that results in a non-significant slope coefficient for the EMG amplitude vs. time relationship and the lowest power output that results in a significant positive slope coefficient [61]. Short term exercise increases the fatigue threshold [62]. By means of the PWCFT heavy from severe domains of exercise intensity can be differentiated [63]. Helpful in this respect are also the power output associated with the gas exchange threshold (PGET), the respiratory compensation point (PRCP), and the critical power [64, 65]. Usually, aerobic exercise training at PWCFT is carried out as a task to produce EIMUF [66]. The absent correlation between PWCFT, PGET, and MPFFT suggests that different physiological mechanisms underlie these three fatigue thresholds [65].

*Cardiac parameters.* EIMUF is dependent on the muscular blood flow and thus on cardiac function, therefore monitoring and evaluation of basic cardiac parameters can be helpful for assessing EIMUF. Frequently applied cardiac parameters include the heart rate, the post-exercise heart-rate recovery (HRR, rate at which heart rate declines after exercise) [3, 45], and the heart rate variability (LnrMSSD) calculated from the long-term ECG [3, 45]. Heart rate is one of the most common parameters to assess the internal load during exercise [3]. This is because of the linear relationship between heart rate and oxygen consumption during steady state exercise [3]. Since there is a positive correlation between THIR, rating of perceived fatigue, and CMJ, HRR and LnrMSSD are promising candidates for non-invasive BMs of the fatigue status in elite soccer players [45].

Movement kinematics and kinetics. Parameters of movement kinematics or kinetics correlated with changes in the muscle fatigue state as measured by EMG interference pattern [67, 68]. Knee and ankle kinematics can be recorded via optical motion capture [11, 69]. For example, kinematics and kinetics during a CMJ or drop jump can be measured by means of a 9 camera motion analysis system (VICON, 100Hz) and a force plate [47] or the Motion Analysis Corporation 3D kinematic analysis system (200Hz) [37]. Evaluated parameters of movement kinematics (jump height, maximal vertical ground reaction force, reactivity strength index, lower limb joint work) may serve as BMs of EIMUF [47]. Other parameters of kinematic measurements by means of motion tracking systems include the mean and variability of joint angles, joint torque, and joint net movements for the shoulder, elbow, and wrist [69]. These parameters usually decrease during fatigue. Increased kinematic variability may be found in the more proximal muscles and decreased kinematic variability in the more distal muscles [69]. Kinematic and kinetic adaptations during fatigue are regarded as reactions to reduce biomechanical loading [69].

Myo-mechanogram (MMG). A further tool to monitor EIMUF is the MMG, which measures the parameters peak torque, contraction time, relaxation time, the acceleration force development and relaxation, the slope and tau of force relaxation, and the mean power frequency fatigue threshold [65, 70]. Peak torque, acceleration of force development, acceleration of relaxation, slope of force relaxation and tau of force relaxation decrease during EIMUF, while contraction time, relaxation time, and the tau of force relaxation increase [70].

Muscle imaging. Functional analysis of muscles can be easily carried out by ultrasound. Particularly muscle fatigue of the diaphragm can be easily monitored by non-invasive ultrasound. A more elaborate method to monitor muscle fatigue is phosphorus magnetic resonance spectroscopy.

Scales and questionnaires. Numerous scores assessing fatigue during or after exercise are available. A major disadvantage of most of these scores is that the CNS component of muscle fatigue is included in the overall assessment of the performance decline after exercise. Further disadvantages are that they rely on subjective information and that many of these scales and questionnaires are not validated for the different types of exercise. Scores most frequently used to assess EIMUF are the perceived rating of fatigue scale [45], the Borg rating perceived exertion (RPE) scale [3], and the session rating of RPE (RPE multiplied by exercise duration). Others include the delayed-onset muscle soreness protocol, the delayed onset muscle soreness (DOMS) score [43], and the Wingate test using the fatigue index [21]. Disadvantage of the RPE is that it shows diurnal fluctuations with higher values at 17.00 h compared to 7.00 h [50]. A further disadvantage of the RPE is that it is increased during submaximal tasks due to compensatory higher central and peripheral inputs [10]. Additionally, physical and subjective changes in performance are less severe in real sport activities as compared to simulated activity [10]. Other dry BMs could be psychomotor speed, monitoring of sleep quality, or the training impulse (TRIMP) [3].

#### Wet biomarkers

Most of the wet BMs of EIMUF derive from blood and only some from the saliva or urine. The most well-known wet BMs are those originating from ATP depletion, from oxidative stress, from muscle damage, or from immunological compromise [71]. A new candidate of wet BMs is the C-terminal agrin fragment. Recently, it became feasible to measure wet BMs directly in the saliva of probands by application of mass spectroscopy [72].

##### ATP metabolism

Depletion of ATP or increase of adenosine diphosphate (ADP) may contribute to EIMUF [2]. The most well-known BMs of EIMUF originating from ATP metabolism include lactate, ammonia, and oxipurines [2].

Lactate. In case the oxidative phosphorylation fails to provide enough ATP for physiologic requirements, generation of ATP shifts from aerobic processes to anaerobic glycolysis or glycogenolysis [73]. A side product of the anaerobic pathway is lactate. Lactate linearly increases with increasing exercise [8]. However, this linear relation is only maintained below the lactate threshold of about 4 mmol/l [74]. Lactate threshold is defined as percentage of maximal workload at which lactate increases above normal. Fatigue develops not earlier than above the lactate threshold due to exponential increase of lactate above 4 mmol/l. A disadvantage of serum lactate is that the relation between fatigue and serum lactate becomes non-linear above the lactate threshold [74]. Further limitations of lactate measurement are that it depends on the ambient temperature, hydration status, diet, lactate clearance rate, glycogen content, previous exercise, and the amount of muscle mass involved in a given exercise [3]. In a study of 12 endurance athletes and 6 power athletes performing fatiguing isokinetic knee flexions/extensions, serum lactate increased with fatigue [52]. Lactate also increased during fatiguing leg press exercise [39]. In a study of 12 healthy subjects, on the contrary, serum lactate decreased after core stabilisation exercise, suggesting that lactate clearance was improved under this condition [75].

Oxipurines (hypoxanthine, xanthine). Oxipurines derive from the degradation of purine nucleotides (adenine, guanine). Oxipurines increase with exercise and are positively correlated with ATP consumption [2]. Oxipurines are a specific and sensitive BM of muscle-cell energy exhaustion during strenuous physical exercise [76]. In a study of 20 healthy males performing isokinetic exercise in a concentric-concentric mode, plasma concentrations of hypoxanthine increased at the end of the exercise [77]. Thus, hypoxanthine was proposed to serve as a BM of work-load effectiveness and of metabolic stress consequences in the muscle [77].

Ammonia. The production of ammonia is triggered by shortage of ATP. In this case, ATP is produced from fusion of two ADPs resulting in one ATP and one AMP. Ammonia originates from the degradation of AMP to IMP and ammonia [2]. Serum ammonia depends on sex. Ammonia increases after short and long duration exercise [2].

##### Increased oxidative stress

BMs of oxidative stress may reflect an increased production of ROS or a decrease of the anti-oxidative capacity. Increased production of ROS results in increased lipid peroxidation or increased protein oxidation. The most well-known BM reflecting lipid peroxidation is TBARS. The most well-known BMs reflecting decreased antioxidative capacity include glutathione (GSH) and total antioxidant capacity (TAC).

Thiobarbituric acid reactive substances (TBARS). TBARS are endproducts of lipid peroxidation, which react with thiobarbituric acid to form a fluorescent red adduct [2, 20]. TBARS are indicators of lipid peroxidation and oxidative stress [2]. TBARS increase with age, physical fitness, and are lower in females as compared to males [2]. TBARS increase 5 min after starting an incremental cycling exercise but increase also 2 days after exercise with maximal strength attributed to macrophage infiltration- and xanthine-oxidase activation-triggered reperfusion of the ischemic muscle [2]. Increase of TBARS during exercise is accompanied by reduced heat shock protein (HSP) production, suggesting that oxidative stress during exercise results from insufficient production of HSP [78].

Glutathione (GSH). GSH is present in nearly all cells but can be also found in blood and the saliva [2]. GSH is one of the most important physiological antioxidants but has other functions as well [20]. As an antioxidant GSH substitutes halogen-, sulphate-, sulphonate-, phosphate- or nitrate groups. GSH levels decrease with age and are higher in females as compared to males. GSH decreases during high-volume training in healthy subjects, which is correlated with a drop in performance. The maximal decrease of GSH during exercise can be observed approximately 5 min after starting exercising [2].

##### Increased oxidative stress

TAC comprises the entire pool of specific and non-specific antioxidants within a cell [20]. TAC includes anti-oxidative enzymes (glutathione peroxidase (GPX), catalase, superoxide dismutase (SOD)), non-specific antioxidants (GSH, ascorbic acid, albumin, uric acid, tocopheroles, carotinoids, coenzyme-Q, bilirubine, and the amino acids cysteine, methionine, tyrosine), and metal chelates. TAC is age- and sex-dependent. TAC is higher in the morning compared to the afternoon [50]. TAC usually decreases during exercise.

Other oxidative stress BMs. Other potential BMs of oxidative stress include catalase [20], protein carbonyls, SOD, isoprostanes, malondialdehyde, uric acid, total bilirubine, and GPX. They have been described in detail elsewhere [2]. A disadvantage of most oxidative stress BMs is that they show diurnal fluctuations with lower values in the evening compared to the morning [50]. Antioxidant parameters at rest are higher in the morning as compared to the evening [21]. Antioxidative efficiency decreases with age and damage from oxidative stress increases with age [71].

##### Inflammatory response

It is well appreciated that particularly long-duration exercise triggers an inflammatory response in the fatiguing muscle. The most frequently applied BMs of this inflammatory response include the leukocyte count, cortisol, and interleukin-6 (IL-6).

Leukocytes. After a marathon, the leukocyte count is increased by 163 %, the thrombocyte count by 20 % and the erythrocyte count by 2.1 % [14]. However, a study on the leukocyte response to bench stepping (high stress) and to repeated eccentric muscle action (low stress) 4, 24, 48, and 72 h after exercise and by assessing perceived muscle soreness by means of the delayed-onset muscle soreness score showed that systemic stress elicited during an acute bout of eccentric exercise has a stronger influence on the functional leukocyte response than the degree of muscle damage induced [79].

Cortisol. Cortisol levels increase after various types of exercise. Salivary free cortisol increases in middle and long-distance runners after the race [80]. Salivary free cortisol correlates negatively with CMJ height after middle-distance run [80].

Interleukin-6 (IL-6). IL-6 is secreted by T-lymphocytes and macrophages to stimulate the immune response after trauma or other tissue damage, leading to inflammation [2]. IL-6 also functions as a myokine, such that it increases in response to muscle contraction [81, 82]. IL-6 mobilises substrates, increases uptake of glucose, hepatic glucose production during exercise, insulin-mediated glucose disposal or lipolysis, and fat oxidation [2]. IL-6 is higher in females as compared to males. IL-6 increases exponentially with exercise. IL-6 is released to the circulation shortly after onset of exercise, precedes the appearance of other cytokines, peaks immediately after exercise, and returns to normal within a few hours after the bout [83]. IL-6 correlates with duration, intensity, number of active muscles, and endurance capacity [2].

Other inflammatory BMs. Other potential inflammatory markers to monitor EIMUF include C-reactive protein [46], TNF-alpha, and other interleukines (e.g. IL-1b, IL-10).

##### Muscle damage

BMs of muscle damage include serum CK, LDH and myoglobin. Additionally, myoglobin can be determined in the urine. These BMs are particularly increased after middle- or long-duration exercise, and are hardly useful for monitoring short-duration exercise. CK and LDH remain increased even 24–72 h after 4 sets of 10 repetition maximum loads for the chest press, pullover, biceps curl, triceps extension, leg extension, and prone leg curl [84]. Neuromuscular function is also compromised for up to 48 h after a Rugby match as evidenced by decreased peak RFD, PP, and PF on the CMJ [44]. CK increased 30 min after a rugby match with a maximum 24 h later and remaining elevated for 120 h [44]. BMs of muscle damage show diurnal fluctuations with higher values in the evening compared to the morning [50]. Damage to muscle tissue after a Rugby League match persists for at least 5 days post-match [44]. Disadvantage of CK is that the temporal relationship with muscle recovery is poor [3].

##### C-terminal agrin fragment (CAF)

Agrin is a protein of the neuromuscular junction. Its activity is regulated by neurotrypsin, which cleaves it into the C-terminal agrin fragment (CAF). If there is excessive neurotrypsin activity degeneration of the neuromuscular junction and thus decreased power output ensues [61]. In a study of 22 healthy older subjects the CAF was determined from blood [61]. Additionally, EMG-signals were recorded at PWCFT during a 2 min discontinuous incremental cycle ergometer exercise [85, 86]. Recorded EMG signals were converted as RMS. In men there was a negative correlation between CAF and PWCFT [61]. It was concluded that CAF concentrations are related to onset of muscle fatigue (only in men) irrespective of age and BMI [61].

##### Other potential wet BMs

Other potential BMs for monitoring EIMUF include testosterone, salivary immunoglobulin-A, natural killer cell activity, neutrophil phagocytic activity, methyl-histidine, glucose-1-phosphate, glucose-6-phosphate, and taurine, of which some can be easily measured in the saliva by means of capillary electrophoresis and time-of-flight mass spectroscopy (CE-TOFMS) [46, 72].

#### Volatile BMs

To sustain endurance performance of the muscle the ability to deliver oxygen to the muscle and the ability of the muscle to utilise a given oxygen load for a sustained period of time (respiratory exchange rate) are important [87]. Maximal oxygen uptake (VO_2_ max) as measured by breath-by-breath open-circuit spirometry is thus a well-established parameter to assess muscle performance [88]. VO2 max decreases with EIMUF but decreases also during age [89]. VO_2_ max is defined as highest VO_2_ value when at least 2 of 3 criteria are met: 1. a plateau of the heart rate or a heart rate within 10 % of the age-predicted maximal heart rate, 2. A plateau of VO_2_, or 3. A respiratory exchange rate >1 [88]. Ventilatory threshold is defined as intersection of 2 regression lines (V_E_ and VO_2_) [88]. Recently, it has been proposed that slow VO_2_ kinetics should be regarded as a volatile BM of exercise intolerance [90]. Slow VO_2_ kinetics after exercise is correlated with greater PCr decrease in the cytoplasm [90]. Conversely, small PCr decrease is strictly correlated with fast VO_2_ kinetics [90]. Putative volatile BMs of EIMUF other than VO_2_ could be the VCO_2_, and the respiratory exchange ratio.

#### Combinations of BMs

Two or more wet BMs may be combined or two or more dry BMs may be combined for monitoring EIMUF. It is also possible to mix wet, volatile, and dry BMs in a variable ratio of components.

##### Real-time fatigue monitoring system

Recently, a real-time fatigue monitoring system has been introduced which quantifies EIMUF during cycling at a constant speed of 60 RPM [91]. The system was equipped with a fatigue progression measure, which synchronously recorded surface EMG signals of the lateral vastus and gastrocnemius muscles in one leg as well as cycling speed in a real-time mode [91]. Additionally, cycling velocity, cycling time, kinesiological data, heart rate and Borg RPE scale values were taken each minute [91]. From these measures the fatigue progression measure was calculated to measure onset-time and progression of EIMUF [91]. The online fatigue monitoring system was validated in healthy subjects and met the intended purposes.

##### Fatiguing leg press exercise

Static fatiguing leg press exercise until exhaustion resulted in increased blood lactate and increased MRV of the agonist and antagonist muscles [39]. Simultaneously, mean power output and MF of surface-EMG decreased in agonist and antagonist muscles [39]. Shift to lower frequencies, increase of MRV, and accumulation of lactate were regarded as independent BMs of fatigue [39].

## Discussion

This review shows that there is progress in the development of BMs to monitor EIMUF, that there are different types of EIMUF depending on the individual, type, intensity, and duration of exercise, and the environmental conditions, that there is a huge number of putative wet, volatile, and dry BMs to monitor EIMUF, and that there is controversy if a single BM or multiple BMs are more suitable for monitoring EIMUF [1]. This review also shows that EIMUF is due to different simultaneously active mechanisms and that the amount of contribution of these different mechanisms changes with type, intensity, and duration of exercise. The choice of a particular BM may additionally be influenced by sex, ethnicity, climate, and daytime. Additionally, only few BMs have strong scientific evidence supporting their use, and there is yet to be a single definite BM of EIMUF described in the literature [3]. A single definite tool of BMs that is accurate and reliable is not currently evident [3]. Currently, there is no gold standard for monitoring the various physiological alterations arising in the skeletal muscle during EIMUF. This is why we recommend to analyse several BMs simultaneously and to further evaluate known BMs and to search intensively for new BMs. Future work should be particularly directed towards the question which combination of BMs is most suitable to monitor EIMUF from a specific type of exercise in a specific group of probands.

Disadvantages of most BMs currently available are that appropriate reference limits are available only for a single given task and that BMs are frequently not validated in general and particularly for other tasks. Since reference limits for BMs for physically active subjects or athletes are lacking, there is a need to establish such reference limits adapted to different types of exercise [1]. Each BM needs to be validated for each type of exercise, to find out which pattern of BMs is most suitable for monitoring EIMUF during or after a particular exercise mode. Additionally, the effectivity of a particular BM for monitoring muscle fatigue needs to be investigated and validated for all types of exercise. If a BM is sufficiently validated, it can be used for monitoring performance and progress in training, identifying overtraining, health-related aspects, which can be modified by regular physical activity, or the physiological response to exercise in healthy and diseased subjects, professional athletes, or amateur athletes. In sport medicine BMs may be helpful to identify subjects who are at an increased risk of poor adaptation to training.

The choice of BMs for monitoring EIMUF is much dependent on the type of exercise to be monitored. Static exercise allows applying more complex and invasive BMs also directly during the workout whereas dynamic exercise allows only determining and monitoring online core temperature, heart rate, and speed. EIMUF from dynamic exercise can be more easily monitored by tests carried out before and after the workload. As an example, electrophysiological techniques are hardly applicable during dynamic exercise and remain the domain of static exercise induced fatigue. Wet BMs also remain the domain of static exercise with determination before and after the bout since measurement during dynamic exercise is only possible by interrupting the task for collecting the tissue of interest. The most intensively investigated and most frequently applied BMs of EIMUF are wet BMs, which include markers of ATP depletion, oxidative stress, of muscle damage, and inflammatory markers. Increasingly investigated are also dry BMs since they are usually more easily obtained and less invasive. Other factors that determine which BMs should be used in a specific study derive from the examiner’s end ( availability of special equipment , the expertise of the researchers in using it, how time-consuming the technique is) and from the examined individuals’ end ( ability to perform a specific task with a specific disease or in a specific age group).

## Conclusions

This review shows that some well-known BMs are available and promising BMs have been recently introduced to monitor EIMUF although the pathophysiology of EIMUF remains to be elucidated. To apply a single BM or a combination of BMs for monitoring EIMUF it is essential that their reliability and applicability is validated in appropriate studies on healthy and diseased, trained and untrained, old and young subjects using all different types of exercise. Currently, it appears that a combination of BMs reflects the various different fatigue mechanisms during a certain exercise more adequately than a single BM. The most appropriate sets of BMs for a given task, however, remain to be ascertained and to achieve scientific legitimacy.

## Abbreviations

ADP: Adenosin-diphosphate
ALS: Amyotrophic lateral sclerosis
AMP: Adenosin-monophosphate
ATP: Adenosin-triphosphate
BM: Biomarker
CAF: C-terminal agrin fragment
CE-TOFMS: Capillary electrophoresis and time-of-flight mass spectroscopy
CK: Creatine-kinase
CMJ: Counter-movement jump
CNS: Central nervous system
COPD: Chronic obstructive pulmonary disease
CSI: Cardiac stress index
DFA: Detrended fluctuation analysis
DOMS: Delayed onset muscle soreness
EIMUF: Exercise-induced muscle fatigue
EMG: Electromyography
GPX: Glutathione peroxidase
GSH: Glutathione
HRR: Heart-rate recovery
HSP: Heat shock protein
IL: Interleukine
IMP: Inosine monophosphate
LDH: Lactate dehydrogenase
LnrMSSD: Heart rate variability
MD1: Myotonic dystrophy 1
MF: Mean frequency
MFCV: Muscle fiber conduction velocity
MID: Mitochondrial disorder
MIFGC: Maximum isometric force-generating capacity
MMG: Myo-mechanogram
MRV: Mean rectified voltage
MVC: Maximal voluntary contraction
OPMD: Oculo-pharyngeal muscular dystrophy
PAP: Postactivation potentiation
PF: Peak force
PGC: Transcriptional coactivator peroxisome-proliferator-activated receptor alpha
PGET: Power output associated with the gas exchange threshold
PP: Peak power
PPAR: Peroxisome proliferator-activated receptor
PRCP: Respiratory compensation point
PRF: Perceived rating of fatigue
PWCFT: Physical working capacity at fatigue threshold
RFD: Rate of force development
RMS: Root mean square
ROS: Reactive oxygen species
RPE: Rating perceived exertions
RSA: Repeated sprint ability
SBMA: Spino-bulbar muscular atrophy
SOD: Superoxide dismutase
TAC: Total antioxidant capacity
TBARS: Thiobarbituric acid reactive substances
THIR: Total high-intensity running distance
TMS: Transcranial magnetic stimulation
TRIMP: Training impulse
UMTT: University of Montreal track test
